# Prediction of Length of Stay Following Elective Percutaneous Coronary Intervention

**DOI:** 10.5402/2011/714935

**Published:** 2011-07-18

**Authors:** Abdissa Negassa, E. Scott Monrad

**Affiliations:** ^1^Division of Biostatistics, Department of Epidemiology and Population Health, Albert Einstein College of Medicine, 1300 Morris Park, Mazer 220, Bronx, New York, NY 10461, USA; ^2^Division of Cardiology, Department of Medicine, Montefiore Medical Center, Albert Einstein College of Medicine, New York, NY, USA

## Abstract

There have been published risk stratification approaches to predict complications following percutaneous coronary interventions (PCI). However, a formal assessment of such approaches with respect to predicting length of stay (LOS) is lacking. Therefore, we sought to assess the performance of, an easy-to-use, tree-structured prognostic classification model in predicting LOS among patients with elective PCI. The study is based on the New York State PCI database. The model was developed on data for 1999-2000, consisting of 67,766 procedures. Validation was carried out, with respect to LOS, using data for 2001-2002, consisting of 79,545 procedures. The risk groups identified by the model exhibited a strong progressively increasing relative risk pattern of longer LOS. The predicted average LOS ranged from 3 to 9 days. The performance of this model was comparable to other published risk scores. In conclusion, the tree-structured prognostic classification is a model which can be easily applied to aid practitioners early on in their decision process regarding the need for extra resources required for the management of more complicated patients following PCI, or to justify to payors the extra costs required for the management of patients who have required extended observation and care after PCI.

## 1. Introduction

A number of models predicting prognosis after percutaneous coronary intervention (PCI), most of them predicting in-hospital mortality, have been reported [1–16]. Such models are valuable in optimizing care for patients undergoing PCI. They can assist health care providers, patients, and their families understand the attendant risks of the procedure and provide an objective basis for determining suitable treatment options [17]. Currently, the procedural success rates are high with low-attendant periprocedural complications [18]. Nevertheless, individual patients with high risk profiles and complicated hospital courses continue to need advanced cardiac care*. *Also important are other postprocedural outcomes such as length of stay (LOS). LOS in particular is an index of patient safety and is a driver of health care expenditure [19]. LOS is likely to be influenced by both patient presenting features and procedural complications; therefore, it is logical to assess the performance of postprocedural risk classification models with respect to LOS.

An easy-to-use tree-structured prognostic classification model was shown to perform well in risk stratifying patients undergoing urgent and emergent PCI [15, 16]. Compared to the traditional log-linear models, this alternative modeling approach is more likely to detect nonlinear relationships, lend itself to easier interpretation, and may be better suited for real-time prognostic classification [20]. Therefore, we report herein the application of a tree-structured prognostic classification model with regard to LOS in *elective* PCI setting. Our model is compared with the Mayo Clinic Risk Score [12, 13], and another risk score developed using the New York State Percutaneous Coronary Interventions Reporting System (PCIRS) [14]. 

## 2. Methods

The analysis was based on the New York State PCIRS database which the New York State Department of Health and its Cardiac Advisory Committee established. The PCIRS is the largest state-oriented collection of audited data on patient outcomes from angioplasty nationwide with a standardized data collection form and quality control mechanism [6, 21]. We restricted our analysis to “elective” procedures, as planning consideration for LOS is more relevant in an elective setting. We defined “elective” PCI as all procedures that were not urgent/emergent, that is, PCI not within 24 hrs of MI, and excluding patients with shock and hemodynamic instability. We also defined LOS as the number of days between admission and discharge, with a minimum of a day.

The Mayo Clinic Risk Score (MCRS) for in-hospital complication was derived from a multivariable logistic regression model [12, 13]. In brief, it is computed as the sum of integer scores based upon the following patient's presenting features, age (+1 for each decade after 30), cardiogenic shock (+5), serum creatinine >2.5 mg/dL or renal failure requiring dialysis (+3), being an urgent/emergent procedure (+2), New York Heart Association (NYHA) functional class III/IV heart failure (+2), the presence of angiographic thrombus (+2), left main disease (+5), and multivessel disease (+2). Based on the total risk score, patients were classified into risk categories of very low risk (MCRS = 0–5), low risk (MCRS = 6–8), moderate risk (MCRS = 9–11), high risk (MCRS = 12–14), and very high risk (MCRS ≥ 15). Multivessel disease was defined as the presence of at least one lesion with ≥70% diameter stenosis in at least two vessels. Some discrepancies in definition between the variables used in the original MCRS derivation and those available in the PCIRS database were noted, and these discrepancies were resolved on the basis of practical considerations as described in earlier publication [22]. The MCRS involves both presenting and procedural characteristics. In the current analysis, the contributions to the score from cardiogenic shock and being an urgent/emergent procedure are dropped due to restriction to elective PCI.

The second comparison model, the approach by Wu et al. [14], also employed a multivariable logistic regression model to develop a risk score for in-hospital mortality using the method described by Sullivan et al. [23]. In brief, they computed for each risk factor in their final model distance from its base (reference) category in regression coefficients units. This distance was then divided by a constant, which is equivalent to the increase in risk in regression coefficients units associated with an increase of five years in age, to derive the variables' point score. The variables in their final model, along with the corresponding scores, are shown in Table 1. In their approach, the summary risk score ranges from 0 to 40. Unlike the MCRS, the authors did not provide cutpoints to classify patients into risk categories. However, one of the attractive features of this risk score is that it is based largely on preprocedural characteristics.

A prediction tree is a particular kind of decision tree. The “decision” is to make a specific prediction regarding outcome, say in-hospital complications, given certain demographic and clinical characteristics. In brief, tree construction involves recursively partitioning the data set on the basis of a set of simple binary (yes/no) questions phrased in terms of the covariates. For example, is age ≥70? The algorithm proceeds starting from the whole data set until further partitioning is not possible either because of homogeneity or small size of the resulting subgroups. The final resulting structure is a binary tree [20]. Potentially, it is possible to grow a very large tree with homogeneous or pure subgroups containing very few subjects, hence, leading to overfitting. To avoid the problem of overfitting, cross-validation along with computationally light model selection approach is employed to select the final tree [24, 25]. The subgroups derived from the final tree constitute the *prognostic classes*. For a more detailed treatment of tree growing, we refer the reader to Breiman et al. [20].The statistical summaries characterizing the prognostic classes can be presented using comparative measures such as odds ratios or relative risks. While the development of a tree-structured prognostic classification is computationally intensive and unfamiliar to practitioners, its actual application is far simpler than other alternative models due to its visual representation as a binary tree structure.

Since in the current analysis LOS is defined as >0, a zero truncated model for count data, that is, the negative binomial model was employed [26]. Model comparison was based on the validation/testing data set (i.e., data set restricted to 2001-2002) employing test for comparing nonnested models [27]. For each approach, the estimated relative risk (RR) and associated 95% confidence interval (CI) comparing each prognostic group to the best prognostic group in terms of LOS are presented. A robust variance estimation procedure was employed to account for clustering of patients within providers [28]. In addition, we assessed the performance of the alternative models by considering extended LOS, that is, LOS ≥10 days, as a binary outcome. 

## 3. Results

Table 2 shows the distribution of covariates considered for analysis. The learning and testing data sets were comparable with respect to the distribution of age at admission, gender, heart failure, smoking status, and other comorbidities. The learning data set was used to develop the tree-structured prognostic classification model for postprocedural complications among patients with “elective” procedures. Postprocedural complication was defined as the combined endpoint of in-hospital death, stroke or coronary bypass surgery. In addition, the learning data set was also used to derive cutpoints for the Wu et al.'s risk score. Since our emphasis is validation, in the following, we present results based on the validation/testing data set. 

The average LOS based on the MCRS stratification is presented in Table 3. LOS monotonically increased with higher MCRS. Compared to patients with very low risk, patients classified as moderate, high, or very high risk incurred progressively higher relative risk of longer LOS (Table 4).

In the original reporting of Wu et al.'s approach, cutpoints for risk stratification were not suggested. However, an intensive search of cutpoints on the learning data set did result into groups that are somewhat distinct in their risk profile. Here, we report results of data-driven cutpoints that were subsequently verified on the testing data set: very low risk (score = 0–5), low risk (score = 6–10), moderate risk (score = 11-12), high risk (score = 13–15), and very high risk (score ≥16). As was the case for MCRS, LOS monotonically increased with higher risk groups (Table 3). Compared to patients in the very low-risk group, patients classified as low, moderate, high, or very high risk incurred progressively longer LOS (Table 4).

**Figure 1:**
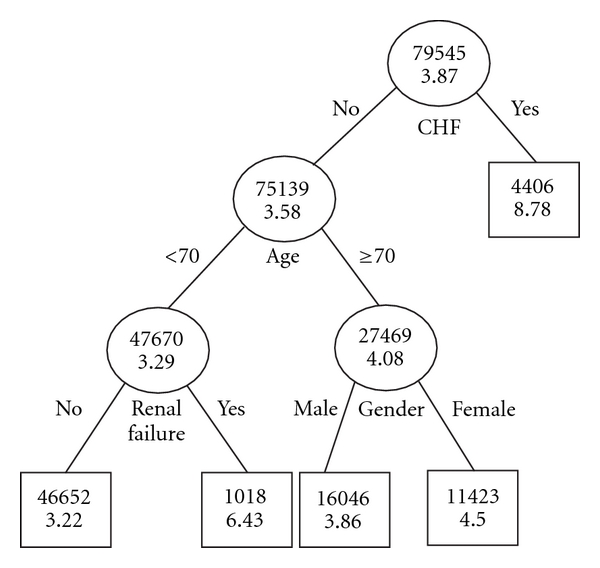
Tree-structured prognostic classification for elective procedures: testing data set. Plain figures are number of procedures in a node, and bold figures are predicted (conditional mean) LOS.

The tree-structured prognostic classification model for predicting risk of complications, among patients undergoing “elective” PCI, identified patients presenting with CHF as very high risk for postprocedural complications. Among patients presenting without CHF, those who were <70 years and with renal failure incurred the next highest risk for postprocedural complications followed by female patients ≥70 years and presenting without CHF. Male patients ≥70 years and presenting without CHF are classified as low risk for postprocedural complications. The remaining patients constituted the best prognostic group (see Figure 1).

LOS progressively increased with the ordering of the above defined prognostic classes (Table 3). Moreover, the prognostic classes are distinct from each other with respect to their LOS experience. Considering the best prognostic class, consisting of patients presenting without CHF and who were <70 years of age and without renal failure, as reference, relative risk for longer LOS, and associated 95% CIs are presented in Table 5.

Defining extended hospital stay (≥10 days) as a binary outcome, the discrimination capacity of the tree-structured prognostic classification and the Wu et al.'s risk score were comparable, 0.734 (95% CI: 0.727, 0.742) and 0.744 (0.737, 0.752), respectively. The MCRS has a slightly lower performance, 0.690 (95%CI: 0.681, 0.698). A further comparison with respect to net-integrated discrimination improvement revealed that the difference between the tree-structured prognostic classification and the Wu et al.'s model was minimal, albeit statistical significance; the integrated discrimination improvement was 0.004 (95% CI: 0.001, 0.007), not clinically significant. 

## 4. Discussion

In this analysis, we applied a novel, tree-structured prognostic classification model as a tool for assessing the risk of longer hospital stay after PCI in patients with elective procedure. This model is based on only four presenting features (heart failure, age, gender, and renal failure) and has a performance that is comparable to the Wu et al.'s risk score [14]. We observed that the risk of longer LOS progressively increases with the ordering of the prognostic classes, that is, from low to very high risk. The extra LOS incurred, on average, ranged from about a day to 6 days. 

The MCRS has been employed extensively [12, 13, 22]. However, in order to determine MCRS, in addition to clinical characteristics such as age, renal insufficiency, heart failure, angiographic characteristics such as presence of angiographically evident thrombus, left main coronary disease and multivessel coronary disease are needed which are not available before angiography. As a result, real-time computation of risk score necessarily is delayed until all such information is available. In addition, while one intuitively expects inclusion of intraprocedural variables to improve LOS prediction, our current analysis shows this not to be the case, at least, for MCRS. The risk score by Wu et al. [14] exhibited a statistically better fit in the current analysis and comparable performance with the tree-structured model. However, this approach is based on a relatively larger number of clinical presenting features and one angiographic characteristic (i.e., left main coronary disease). Therefore, it is likely to be considered burdensome to use in an ongoing fashion in the clinical setting. One has to weigh the small gain in accuracy against ease of implementation. 

By contrast, the tree-structured prognostic classification model utilizes only 4 clinical presenting features that can be readily assessed any time prior to catheterization. This simpler, truly a priori approach enjoys a performance, for all practical purposes, comparable to the approach of Wu et al. With such a large testing data set, very small differences may reach statistical significance. Representation of our model as a binary tree structure enhances its utility in the clinical setting. The need for simplicity in such prediction models has been emphasized in the literature [17]; the proposed tree-structured prognostic classification model meets that need.

The PCIRS database has a rigorous quality control mechanism in place, that is, a standard form is employed for data collection, and accuracy of data is maintained by continuous auditing of medical records [6, 21]. Nonetheless, additional variables that were not available in the database (i.e., STEMI status and glomerular filtration rate) and the way some variables were collected (i.e., the way renal failure was assessed: creatinine >2.5 mg/dL) may have limited the possibility of developing a prediction model with even higher performance and does limit comparison with the recently published National Cardiovascular Data Registry (NCDR) risk score [29].

We restricted our analysis to patients with elective procedure by excluding all patients with urgent/emergent indication for PCI, shock and hemodynamic instability. Our restriction is compatible with the definition of elective PCI adopted by others in the literature [8]. A potential limitation of our study is that the patient mix in the PCIRS database might be different from those treated in other places. However, this database is from a state-wide experience with obvious heterogeneity of patient population and setting. Furthermore, the variables included in our model have been repeatedly shown to be relevant in various databases and studies [14, 29–32].

The tree-structured prognostic classification model allows the efficient and early identification of high-risk groups to facilitate frank discussion of risks and benefits of potential treatment strategies in a clinical setting. Moreover, as length of stay is related to patient morbidity, our approach allows a more accurate consent process with the patient, as more of the relevant risk will be identified preprocedure. This is of an important bioethical consideration, even medical legal. 

Furthermore, our model permits an evidence-based dialogue between the practitioner and the insurance payers about the need (or lack thereof) for admission to hospital after procedure. Hence, enhancing rational, cost-effective, patient care decisions in this regard, and permitting advance planning for a longer than average observation. Similarly, patients deemed to be at low risk might be managed less intensively following PCI, with the possibility of early discharge in order to channel valuable resources to those who need it most. Therefore, the tree-structured prognostic classification model will aid the practitioner, early on, in the decision process regarding the need for extended observation after PCI. This early on assessment should be augmented by the individual patient's specific condition after the procedure, as recommended by a recent consensus panel [19].

In summary, we have shown in this study that the tree-structured model could be employed to determine the risk of a longer length of stay for patients with elective PCI. This approach is easy to use, involves only four presenting features, and has a comparable performance with other risk scores. The model identifies patients with a wide range of anticipated length of stay, that is, on average, up to 9 days. This wide range, potentially, would have an implication in optimal patient management, resource allocation, quality assessment, and utilization reviews. 

## Figures and Tables

**Table 1:** Risk score based on Wu et al.'s approach.

Risk factor	Score
Age (years)	
56–64	1
65–74	3
75 and older	5
Women	1
Hemodynamic state	
Unstable*	6
Shock*	9
Ejection fraction	
<20%	3
20–29%	2
Preprocedural myocardial infarction	
<24 h with stent thrombosis*	9
<6 h without stent thrombosis*	7
6–23 h without stent thrombosis*	6
1–14 days	4
>14 days	2
Peripheral arterial disease	2
Current heart failure	4
Past heart failure	3
Renal failure	
Renal failure, creatinine >2.5 mg/dL	3
Renal failure, requiring dialysis	4
Left main coronary artery disease	3

*Not applicable to “elective” patient population.

**Table 2:** Distribution of covariates by data set.

Covariate	Learning (%) (*N* = 67766)	Testing (%) (*N* = 79545)
Age (Years)	64.1 (11.8)*	64.7 (11.8)
Body mass index	28.7 (5.4)*	28.9 (5.6)
Women	32.1%	32.6%
White	86.9%	83.2%
Black	6.5%	7.3%
Other	6.6%	9.6%
Hispanic	6.5%	7.3%
Current smoker	16.7%	16.9%
Diabetes mellitus	25.8%	28.6%
Hypertension	71.0%	76.2%
Heart failure	5.5%	5.5%
Vascular disease	8.9%	9.4%
Renal failure (including dialysis)	2.3%	2.8%
Chronic lung disease	5.3%	6.1%
Malignant ventricular arrhythmias	1.4%	0.8%
Prior myocardial infarction	6.9%	6.9%
Prior percutaneous coronary intervention	30.6%	35.3%
Prior open heart surgery	18.0%	19.7%
Previous stroke	4.0%	4.3%
Left Main disease	2.7%	2.9%
Multivessel coronary disease	46.0%	47.0%

*Age and body mass index are presented as mean (SD).

**Table 3:** Length of stay (in days) by risk category.

	Risk category				
	Very low	Low	Moderate	High	Very high
Testing data set (%)					
MCRS^†^	18.7%	65.7%	11.3%	3.7%	0.6%
Wu et al.^‡^	53.2%	40.2%	3.2%	2.4%	1.0%
TSPC^§^	58.7%	20.2%	14.4%	1.3%	5.5%

Conditional mean of LOS from corresponding model					
MCRS	3.2	3.5	5.8	7.8	9.4
Wu et al.	3.3	4.3	6.9	9.1	11.1
TSPC	3.2	3.9	4.4	6.4	8.7

Legend: MCRS = Mayo clinic risk score; TSPC: tree-structured prognostic classification.

^†^Sum score for very low (MCRS = 0–5), low (MCRS = 6–8), moderate (MCRS = 9–11), high (MCRS = 12–14), and very high (MCRS ≥15).

^‡^Sum score for very low (0–5), low (6–10), moderate (11-12), high (13–15) and very high (≥16).

^§^Very low = (no heart failure and <70 years and without renal failure), low = (no heart failure and ≥ 70 years and male), moderate = (no heart failure and ≥70 years and female), high = (no heart failure and <70 years and with renal failure), and very high = (heart failure present).

**Table 4:** Relative risk comparing each risk group with the reference group with respect to LOS based on the testing data set.

Risk group	MCRS*	Wu et al.^†^
Very low	1.0	1.0
Low	1.13 (1.10, 1.16)	1.55 (1.50, 1.59)
Moderate	2.11 (2.01, 2.20)	2.64 (2.49, 2.81)
High	2.88 (2.72, 3.06)	3.37 (3.16, 3.60)
Very high	3.56 (3.20, 3.96)	4.42 (4.09, 4.79)
LL^‡^	−170178.25	−168971.91

Legend: MCRS: mayo clinic risk score.

*Sum score for very low (MCRS = 0–5), low (MCRS = 6–8), moderate (MCRS = 9–11), high (MCRS = 12–14), and very high (MCRS ≥15).

^†^Sum score for very low (0–5), low (6–10), moderate (11-12), high (13–15), and very high (≥16).

^‡^LL: log likelihood.

^¶^ *P* = 0.000 comparing MCRS with Wu et al.'s risk score suggests that Wu et al.'s model providing a better fit.

**Table 5:** Relative risk comparing each risk group with the reference group with respect to LOS based on tree-structured prognostic classification.

Risk group	Testing data set
Very low (no heart failure and <70 years and without renal failure)	1.0
Low (no heart failure and ≥70 years and male)	1.26 (1.23, 1.30)
Moderate (no heart failure and ≥70 years and female)	1.48 (1.43, 1.54)
High (no heart failure and <70 years and with renal failure )	2.29 (2.06, 2.54)
Very high (heart failure present)	3.20 (3.05, 3.34)
LL*	−169549.66^†^

*LL: log likelihood.

^†^ *P* = 0.002 comparing tree-structured prognostic classification with Wu et al.'s risk score suggests that Wu et al.'s model providing a better fit.
